# Chemical profile of *Lippia thymoides*, evaluation of the acetylcholinesterase inhibitory activity of its essential oil, and molecular docking and molecular dynamics simulations

**DOI:** 10.1371/journal.pone.0213393

**Published:** 2019-03-08

**Authors:** Sebastião Gomes Silva, Renato Araújo da Costa, Mozaniel Santana de Oliveira, Jorddy Neves da Cruz, Pablo Luis B. Figueiredo, Davi do Socorro Barros Brasil, Lidiane Diniz Nascimento, Antônio Maia de Jesus Chaves Neto, Raul Nunes de Carvalho Junior, Eloisa Helena de Aguiar Andrade

**Affiliations:** 1 Program of Post-Graduation in Chemistry, Federal University of Pará, Belém, PA, Brazil; 2 LABEX/FEA (Faculty of Food Engineering), Program of Post-Graduation in Food Science and Technology, Federal University of Para, Belém, PA, Brazil; 3 Laboratory of Preparation and Computation of Nanomaterials, Federal University of Pará, Belém, PA, Brazil; 4 Department of Natural Sciences, State University of Pará, Belém, PA, Brazil; 5 Program of Post-Graduation in Engineering of Natural Resources of Amazon, Federal University of Pará, Belém, PA, Brazil; 6 Adolpho Ducke Laboratory, Botany Coordinating, Museu Paraense Emílio Goeldi, Belém, PA, Brazil; The University of Alabama, UNITED STATES

## Abstract

The essential oils of the fresh and dry flowers, leaves, branches, and roots of *Lippia thymoides* were obtained by hydrodistillation and analyzed using gas chromatography (GC) and GC–mass spectrometry (MS). The acetylcholinesterase inhibitory activity of the essential oil of fresh leaves was investigated on silica gel plates. The interactions of the key compounds with acetylcholinesterase were simulated by molecular docking and molecular dynamics studies. In total, 75 compounds were identified, and oxygenated monoterpenes were the dominant components of all the plant parts, ranging from 19.48% to 84.99%. In the roots, the main compounds were saturated and unsaturated fatty acids, having contents varying from 39.5% to 32.17%, respectively. In the evaluation of the anticholinesterase activity, the essential oils (detection limit (DL) = 0.1 ng/spot) were found to be about ten times less active than that of physostigmine (DL = 0.01ng/spot), whereas thymol and thymol acetate presented DL values each of 0.01 ng/spot, equivalent to that of the positive control. Based on the docking and molecular dynamics studies, thymol and thymol acetate interact with the catalytic residues Ser203 and His447 of the active site of acetylcholinesterase. The binding free energies (Δ*G*_bind_) for these ligands were -18.49 and -26.88 kcal/mol, demonstrating that the ligands are able to interact with the protein and inhibit their catalytic activity.

## Introduction

Alzheimer's disease is considered one of the major public health problems worldwide and one of the main complications of this pathology is the activity deficit of cholinergic neurons. This fact can be reversed and/or attenuated by elevating the levels of the neurotransmitter acetylcholine in the neuronal synaptic area. The use of cholinesterase inhibitors is an effective therapeutic approach [1]. The inhibitors increase the availability of neurotransmitters by inhibiting their main catalytic enzymes, acetyl- and butyrylcholinesterase, thus diminishing the cholinergic deficit and relieving the symptoms of Alzheimer's patients [2]. The oldest inhibitor of these enzymes is physostigmine, an alkaloid of the shrub *Physostigma venenosum* Balf [3]. Synthetic and semisynthetic inhibitors, such as galantamine, donepezil, tacrine, and rivastigmine, can also be used, but these drugs have disadvantages such as short half-lives and adverse side effects including hepatotoxicity and gastrointestinal irritation [1,4,5]. This has encouraged a search for new inhibitors from natural sources, and some examples are the secondary metabolites present in essential oils [1,4,6–8].

*Lippia thymoides* is a native and endemic Brazilian species with distribution in the states of Bahia and Minas Gerais in the Caatinga and Cerrado types of vegetation [9]. Folk medicine makes use of this plant for the treatment of wounds, and the leaves are used as an antipyretic and digestive, as well as in the treatment of bronchitis and rheumatism [10,11].

The objective of this study was to obtain and analyze the chemical composition of the essential oils from different parts of *L*. *thymoides* and to evaluate the anticholinesterase potential of the oil of the fresh leaves and its main constituents(thymol and thymol acetate), as well as to evaluate the interactions of acetylcholinesterase (AChE) with thymol and thymol acetate by molecular docking and molecular dynamics simulations and free energy calculations using the molecular mechanics generalized Born surface area (MM/GBSA) method.

## Materials and methods

### Plant material and essential oil extraction

The authors declare that no specific permissions were required for these locations/activities; and we confirm that the field studies did not involve endangered or protected species.

*L*. *thymoides* was collected in the Municipality of Abaetetuba, Eastern Amazon, State of Pará, Brazil. The botanical identification was made by comparison with authentic samples and incorporated into the "João Murça Pires" herbarium of the Museu Paraense Emilio Goeldi (Belém, Pará, Brazil) under catalog number *MG 213373*.

The essential oils were obtained from fresh and dried parts of *L*. *thymoides*.The drying of the plant material was conducted in a forced air convection oven for two days at 34°C. After that the material was ground and submitted to hydrodistillation.

### Hydrodistillation

The extraction of the essential oils of fresh flowers (FFL, 10g), leaves (FLE, 40g), branches (FB, 40g), and roots (FR, 40g), and dried flowers (DFL, 10g), leaves (DLE, 40g), branches (DB, 40g), and roots (DR, 40g) was carried out by hydrodistillation in a Clevenger apparatus for 3 h. Subsequently, the oils were submitted to centrifugation, dehydrated with anhydrous Na_2_SO_4_ and stored at 8°C in a freezer. The moisture content was determined using a moisture analyzer (ID50, Marte) at the moment of extraction. The yield of essential oil (%) was calculated and is expressed as milliliters / 100-g of dried material [12].

### Chemical constituent identification

The chemical composition was determined according to the approach described by Da Silva et al. [13], where the qualitative analyses of essential oils was carried using a gas chromatography–mass spectrometry (GC-MS) Thermo Focus DSQ-II system under the following the operating conditions: DB-5MS silica capillary column (30 m × 0.25 mm; 0.25 μm film thickness), temperature program: 60–250°C with a gradient of 3°C/min); injector temperature: 240°C, helium carrier gas (linear velocity of 32 cm/s, measured at 100°C), injection type: split less (0.1 μL of a 2:1000 *n*-hexane solution),and ion-source temperature and other parts: 200°C. The ionization was achieved by electron impact at 70 eV. Quantitative sample data were obtained using a GC with a flame ionization detector in a Focus GC-FID, which was operated under the same conditions as the GC-MS, except for the carrier gas, which was nitrogen. The identification of volatile components was based on the linear retention index (IR), which was calculated in relation to the retention times of a homologous series of *n*-alkanes and the fragmentation pattern observed in the mass spectra by comparison with authentic samples from the libraries of the data system and the literature [14,15].

### Acetylcholinesterase assay

The AChE assay was conducted according to the method reported by Marston et al. [16] The enzyme AChE (500 U), from Electrophorus electric us (electric eel, Sigma Aldrich, Missouri, EUA, E.C. 3.1.1.7), was dissolved in tris-hydrochloric acid buffer (pH 7.8) and stabilized by the addition of bovine serum albumin fraction V (0.1%). Thymol, thymol acetate, and the essential oil of *L*. *thymoides* fresh leaves were applied to thin-layer chromatography (TLC) plates to obtain spots with concentrations from 0.01 to 1000 ng/spot. Physostigmine was used as a positive control. The plates were sprayed with the AChE solution (3.33 U/mL), dried, and incubated at 37°C for 20 min. The enzyme activity was detected by spraying with a solution of 0.25% of 1-naphtyl acetate in ethanol and a 0.25% aqueous solution of Fast Blue B salt (20 mL). Potential acetylcholinesterase inhibitors appeared as clear zones on a purple background.

### Semisynthesis of thymol acetate

To obtain thymol acetate, thymol (Sigma–Aldrich), acetic anhydride, and pyridine, which acts as a catalyst, were used. Thymol (4g) was acetylated with acetic anhydride in the presence of pyridine for 24 h at 25°C. The excess acetic anhydride was removed by storage of the sample in a desiccator for 12 h. The reaction mixture was partitioned with dichloromethane and water for the removal of acetic acid. The semi synthesis and identification of the pure compound was monitored by GC and GC-MS analysis.

### Molecular docking

To analyze the interactions between the ligands (thymol and thymol acetate) and AChE, molecular docking simulations were carried out using the Molegro Virtual Docker (MVD) 5.5 program [17]. The crystallographic structure of AChE was obtained from the Protein Data Bank (https://www.rcsb.org/) (PDB code: 1C2B) [18]. Both the structures of the enzyme and the ligands were prepared using the MVD module. Before the docking simulation, for each complex, hydrogen atoms and partial atomic charges were added. The active site of AChE was positioned in a spherical grid of 10-Å diameter, and all residues of AChE binding site were included using the following spatial coordinates of the central cavity: *x* = 26.40, *y* = 79.03, and *z* = 20.20. A grid resolution equal to 0.3 Å was used. Molecular docking was performed using the standard MolDock algorithm in MVD.

### Molecular dynamics (MD) simulations

Molecular dynamics (MD) simulation for all ligand–protein systems obtained after docking were performed to evaluate possible conformational changes in the protein structure. First, the partial atomic charges were calculated using Gaussian09 [19], using the restrained electrostatic potential (RESP) protocol at the HF/6-31G* level of theory [20,21]. The antechamber [22] module of Amber 16 package was used to parameterize the ligand [23,24], which was described by the General Amber Force Field (GAFF) [25].

The protonation state of all ionizable residues was determined by the PDB2PQR server [26,27], and the protein structure was treated with the ff14SB force-field [28] in all MD simulations. The protein system was solvated in octahedral periodic box with the TIP3P explicit solvation model [29]. Counterions were added to neutralize the system charges. The system contained approximately 16,200 water molecules and 55,500 atoms. A cut-off distance of 12 Å and the particle mesh Ewald method [30] was used for electrostatic calculations, and the SHAKE algorithm was used to keep all the hydrogen bonds at their pre-defined equilibrium distances during minimization [31]. Before the MD simulation, production systems underwent simulations for energy minimization, heating, and equilibration that were performed with the Sander module and pmemd. CUDA [32].

Energy minimization was performed in two steps. In each step, 1,000 steepest descent and conjugate gradient algorithm cycles were applied. In these steps, bad contacts and possible steric conflicts were removed, and the protein system acquired the most energetically favorable conformational state. First, the protein structure was restrained with a harmonic force constant of 100 kcal/mol Å^−2,^ and the water molecules and counterions were not treated with harmonic restraints. Then, the harmonic constraint was removed to perform the MD run of the protein system (protein, water, and counterions). This system was then heated to 300 K in five steps for 500 ps. In the first four steps, we applied a harmonic force constant of 25 kcal/mol Å^−2^ to restrain all heavy atoms. In the last heating step, the harmonic force constraint was removed. The Langevin thermostat [33] within a collision frequency of 2 ps^−1^was used to maintain the temperature. Thus, 5 ns of MD simulation without harmonic restraints at 300 K was carried out to obtain system equilibrium. Finally, a 100-ns MD simulation was performed for all protein systems.

### Binding free energy calculations using the MM-GBSA approach

The molecular mechanics–generalized Born surface area (MM-GBSA) method [34,35] was used to measure the binding free energies of the ligands, thymol and thymol acetate. The binding free energies (Δ*G*_bind_) were calculated according to the following equations:

$${\Delta G}_{\mathrm{bind}}={\Delta G}_{\mathrm{complex}}‑{\Delta G}_{\mathrm{receptor}}‑{\Delta G}_{\mathrm{ligand}}$$ (1)

Each free energy state is calculated by the following equations:

$${\Delta G}_{\mathrm{bind}}=\Delta H‑T\Delta S\approx {\Delta E}_{\mathrm{MM}}+{\Delta G}_{\mathrm{solv}}‑T\Delta S$$ (2)

$${\Delta E}_{\mathrm{MM}}={\Delta E}_{\mathrm{internal}}+{\Delta E}_{\mathrm{ele}}+{\Delta E}_{\mathrm{vdW}}$$ (3)

$${\Delta G}_{\mathrm{solv}}={\Delta G}_{\mathrm{GB}}+{\Delta G}_{\mathrm{SA}}$$ (4)

The Δ*G*_bind_ values correspond to the sum of interaction energies in the gas phase between the protein and ligand (Δ*E*_MM_), the desolvation free energy (Δ*G*_solv_), and the system entropy (−*T*Δ*S*). Here, Δ*E*_MM_ is the sum of internal energy (Δ*E*_internal_), sum of bond length, angle, and dihedral energies), electrostatic contributions (Δ*E*_electrostatic_), and van der Waals terms (Δ*E*_vdW_). Δ*G*_solv_ is the sum of the polar contributions (Δ*G*_GB_) and non-polar contributions (Δ*G*_SA_). The external dielectric constant of the solute was defined as 80, while the internal dielectric constant was defined as 1. Δ*G*_SA_ was determined from the solvent accessible surface area (SASA) estimated using the LCPO algorithm. For the free energy calculations, 1,000 MD frames were used, corresponding to the last 5 ns of the simulations.

### Per-residue binding free energy decomposition

To analyze the free energy contributions of each amino acid residue of protein pocket for ligand interaction, the binding free energy was decomposed into the van der Waals (Δ*E*_vdW_) and electrostatic (Δ*E*_electrostatic_) contributions in the gas phase, as well as the polar solvation (ΔG_pol_) and nonpolar solvation (Δ*G*_nonpol_) contributions [36], using the following equation:

$${\Delta G}_{\mathrm{inhibitor}‑\mathrm{residue}}={\Delta E}_{\mathrm{vdW}}+{\Delta E}_{\mathrm{ele}}+{\Delta G}_{\mathrm{pol}}+{\Delta G}_{\mathrm{nonpol}}$$ (5)

## Results and discussion

### Yield and composition of essential oil

The yield of *L*. *thymoides* essential oil varied in quantity in the different plant parts and with treatment (fresh and dried). The flowers produced the highest essential oil yields, both fresh and dried (FFL 5.8%; DFL 7.3%). DB presented an oil yield equal to 0.14%, whereas FB, FR, and DR contained only traces of essential oil (Table 1).

**Table 1:** Essential oil composition of different organs of *Lippia thymoides*.

Oil Yield		FFL	DFL	FLE	DLE	FB	DB	FR	DR
		5.80	7.30	1.87	1.25	tr	0.14	tr	tr
RI	Constituents	Composition %							
904	1-ethylbutyl hydroperoxide							0.28	0.21
914	1-methylpentyl hydroperoxide							0.32	0.20
923	α-thujene		1.46	0.24	0.81	0.24	0.26		
933	α-pinene		0.20						
947	Camphene		0.11	0.02	0.05	0.03			
969	Sabinense		0.21	0.04		0.08	0.04		
974	β-pinene		0.05	0.04	0.18	0.10	0.12		
989	Myrcene		1.67	0.68	1.34	0.43	0.60		
1003	α-phellandrene		0.30	0.10	0.13	0.07	0.08		
1016	α-terpinene	0.02	1.99	0.55	1.48	0.57	0.77		
1022	*p*-cymene	0.07	7.18	5.30	8.36	3.27	3.35		
1026	Limonene		0.28	0.15	0.23	0.16	0.14		
1028	1,8-cineole	0.19	0.30	0.39	0.48	0.24	0.30		
1045	(E)-β-ocimene		0.13	0.10	0.11	0.06	0.10		
1057	γ-terpinene	0.15	15.06	7.58	9.36	3.39	4.84		
1068	cis-sabinene hydrate	0.14	0.12						
1089	Terpinolene		0.10	0.06	0.09		0.06		
1092	*para*-cymenene	0.02	0.04	0.03	0.04				
1097	Linalool	0.08	0.07	0.14	0.16		0.15		
1100	trans-sabinene hydrate	0.03	0.04						
1143	Camphor	0.10	0.10	0.10	0.12	0.06	0.08		
1147	3-methyl-3-butenyl-3-methylbutanoate	0.06	0.05	0.02	0.02				
1166	Borneol		0.03	0.06	0.05				
1169	Umbellulone	0.44	0.23	0.02	0.03	0.32	0.33		
1175	terpinen-4-ol	0.32	0.37	0.29	0.52	0.41	0.56	0.07	
1188	α-terpineol	0.02	0.02	0.03			0.03		
1233	ether methyl thymol	1.82	2.00	1.01	1.27	1.47	1.39	0.07	
1293	Thymol	48.04	37.86	66.33	58.9	63.59	66.20	19.34	22.18
1352	thymol acetate	33.81	21.44	7.49	8.10	5.07	5.96		
1358	Eugenol			0.09	0.08	0.12		0.49	
1372	α-copaene	0.04	0.03		0.03		0.06		
1373	carvacrol acetate					0.05			
1386	β-bourbonene					0.04	0.09		
1401	Methyleugenol						0.03		
1419	β-caryophyllene	9.55	5.93	5.32	4.53	1.29	4.16		
1432	*trans*-α-bergamotene	0.15	0.11	0.16	0.10	0.07	0.13		
1433	γ-elemene	0.06	0.03						
1441	Aromadendrene			0.04	0.03		0.05		
1454	α- humulene	1.35	0.69	0.73	0.61	0.26	0.71		
1479	γ-muurolene	0.16	0.05	0.13	0.12	0.09	0.15		
1486	germacrene-D	1.22	0.70	0.26	0.65	0.42	0.61		
1495	γ-amorphene	0.04		0.05	0.05	0.02	0.07		
1496	α-selinene			0.04		0.04	0.03		
1499	α-muurolene	0.03		0.05	0.04	0.03	0.08		
1510	δ-amorphene				0.02		0.06		
1514	γ-cadinene	0.12	0.05	0.10	0.09	0.02	0.13		
1521	δ-cadinene	0.23	0.10	0.20	0.16	0.08	0.24		
1521	*trans*-calamenene			0.03		0.02	0.05		
1535	*trans*-cadina-1,4-diene			0.02			0.02		
1539	α-cadinene			0.02	0.02		0.02		
1562	germacrene B			0.04	0.03	0.02	0.05		
1579	Spathulenol					0.02			
1583	caryophyllene oxide	0.61	0.29	0.40	0.33	0.66	0.42		
1609	humulene epoxide II	0.05	0.03	0.04	0.03	0.07	0.04		
1637	epi-α-cadinol	0.02	0.02	0.05			0.09		
1641	epi-α-muurolol					0.02			
1645	α-muurolol					0.03	0.03		
1653	α-cadinol	0.07	0.04	0.07	0.05	0.14	0.16		
1663	tetradecanoic acid							0.35	0.33
1668	14-hydroxi-9-epi-(E)-caryophyllene	0.03		0.03	0.03	0.09	0.03		
1862	pentadecanoic acid							0.35	1.15
1900	Nonadecane							0.37	0.72
1920	2-ethylhexyl-3-(4-methoxyphenyl)-2-propenoate								2.04
1955	(11Z)-11-hexadecenoic acid							3.67	2.73
1961	hexadecanoic acid	0.16	0.06			6.11	2.77	40.92	38.02
2080	(9Z,12Z)-9,12-octadecadienóic acid							4.49	
2158	(9Z)-octadecenoic acid					6.26	1.58	28.21	27.40
monoterpene hydrocarbons		0.26	28.78	14.89	22.18	8.40	10.36		
oxygenated monoterpenes		84.99	62.58	75.86	69.63	71.21	75.00	19.48	22.18
sesquiterpene hydrocarbons		12.95	10.69	7.19	6.48	2.41	6.71		
saturated fatty acids		0.16	0.06			6.11	2.77	41.62	39.50
unsaturated fatty acids						6.26	1.58	36.37	32.17
Others		0.84	0.43	0.7	0.54	1.15	0.80	1.46	1.13
**Total**		**99.20**	**99.54**	**98.64**	**98.90**	**95.53**	**97.20**	**98.93**	**96.05**

FFL: fresh flower; DFL: dried flower; FLE: fresh leaves; DLE: dried leaves; FB: fresh branche; DB: dried branche; FR: fresh root; DR: dried root. tr: traces

Generally, the essential oil yield of a plant varies depending on the part, seasonality, and geographical distribution, among other factors. For example, samples of *L*. *citriodora* produced different oil yields depending on the part extracted, and it was verified that the highest yields were obtained from the flowers [37]. Low oil yields from branches and roots (0.1% for both) were also registered in a species of domesticated *L*. *muliflora* [38]. The yield of dried leaves of *L*. *thymoides* from state of Bahia (Brazil) showed seasonal variation, and its values varied between 2.14% and 2.93% [39].

Seventy-five components were identified in the oil, comprising (95.53–99.54%) of the total composition (Table 1). Oxygenated monoterpenes were identified in all plant organs, and the percentage contents of this group varied from 19.48% (FR) to 84.99% (FFL)

The monoterpene and sesquiterpene hydrocarbons varied respectively from 0.26% (FFL) to 28.78% (DFL) and 2.41% (FB) to 12.95% (FFL), but these were absent in the essential oils of the roots. Saturated fatty acids were also identified, whose content varied from 0.06% (DFL) to 41.62% (FR), but these were absent in the leaves; unsaturated fatty acids were detected only in the branches and roots, ranging from 6.26% (FB) to 36.37% (FR).

Thymol was the only constituent present in all the organs of *L*. *thymoides* and was the main component of the oils of the flowers, leaves, and branches. This oxygenated monoterpenoid varied among all the plant organs from 19.34% (FR) to 66.33% (FLE). In the three parts where thymol was the main component. In addition, for the fourth plant sample, quantitative variations in *p*-cymene (0.07% FFL and 8.36% DLE), β-caryophyllene (1.29% FB and 9.55% FFL), γ-terpinene (0.15% FFL and 15.06% DFL), and thymol acetate (5.07% FB and 33.81% FFL) were observed.

The essential oils from the roots were characterized, and hexadecanoic (palmitic) acid was found to be the main component (38.02% DR; 40.92% FR), followed by (9Z)-octadecenoic (oleic) acid (27.4% DR; 28.21% FR), thymol (19.34% FR; 22.18% DR), (9Z,12Z)-9,12-octadecadienoic (linoleic) acid (4.49% FR), and (11Z)-11-hexadecenoic acid (2.73% DR; 3.67% FR). It is important to note that hexadecanoic (palmitic) acid was also present in the oils obtained from the branches (2.77% DB; 6.11% FB) and flowers (0.06% DFL; 0.16% FFL), and(9Z)-octadecenoic (oleic) acid was also identified in the oils of the branches (1.58% DB; 6.26% FB). Please refer to S1 and S2 Figs with the chromatogram ions for the chemical composition of the fractions of essential oils.

Silva et al. [39]reported that the essential oil from the leaves of a specimen of *L*. *thymoides*contains sesquiterpeneβ-caryophyllene (17.22–26.27%) as the main component, followed by borneol (4.45–7.36%), camphor (3.22–8.61%), camphene (2.64–5.66%), and germacrene-D (4.72–6.18%). This chemical profile is different from described in the present study. This is possible related to biotic and abiotic factors of the collection site of these two specimens; according to Ribeiro et al. [40], these factors qualitatively and quantitatively affect the yield and composition of secondary metabolites.

Studies with species of the same genus, such as *L*. *multiflora* Moldenke, showed that the essential oils obtained from the leaves, flowers, branches, and roots do notvary in chemical profile, and the composition was essentially dominated by monoterpenes [38]. Similarly, the volatiles of the vegetative parts (leaves and branches, flowers, and fruit) of *Lippia citriodora* were characterized as having the same constituents in all parts of the plant: geranial (30.67–36.87%), neral (21.71–28.33%), and limonene (6.07–7.27%) [37].

Species of other genera, such as *Hertia cheirifolia*, showed significant variation in the composition of the essential oil between the different parts of the plant, where the flower buds and flowers were characterized by a higher content of drimane-type sesquiterpenes (34.08% and 32.55%, respectively), while the leaves and fruits predominantly contained α-pinene (35.63% and 33.17%, respectively) [41]. In addition, the essential oils of the roots, leaves, branches, inflorescences, and fruits of *Kelussia odoratissima* Mozaff. allcontained the same major compounds: (Z)-ligustilide (54.0–86.0%) and (2e)-decen-1-ol (2.0–12.3%). However, among the samples of these parts, a complex mixture of up to thirty-two different chemical compounds was identified [42].

### Obtaining thymol acetate

**Fig 1:**
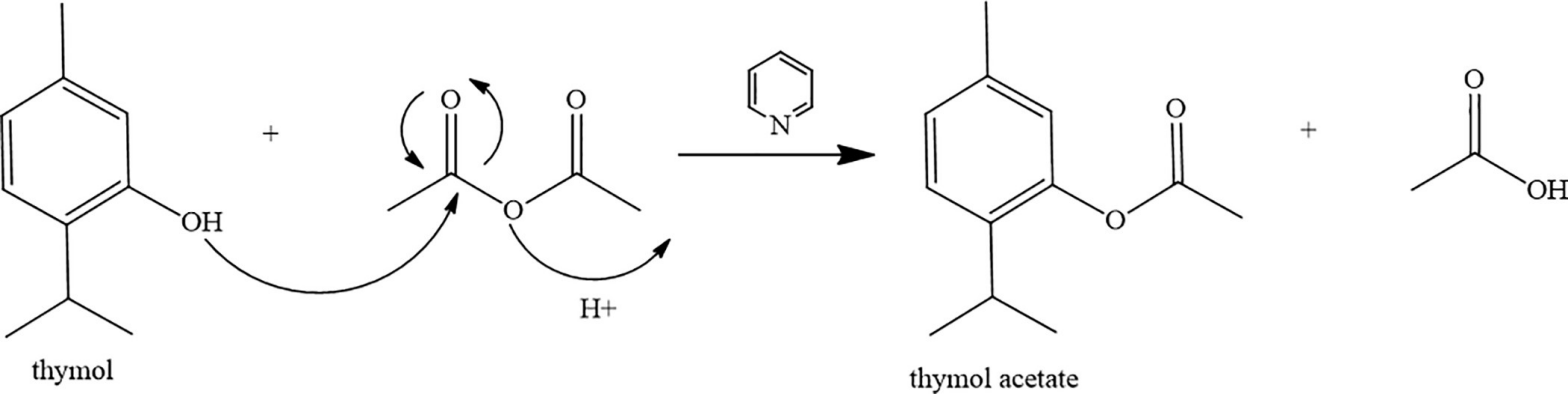
Synthesis reaction of thymol acetate.

**Fig 2:**
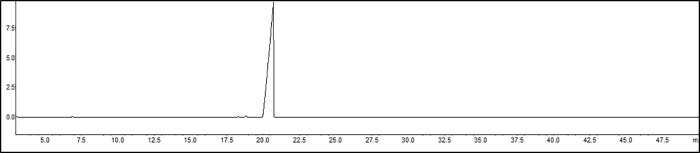
Thymol acetate chromatogram.

**Fig 3:**
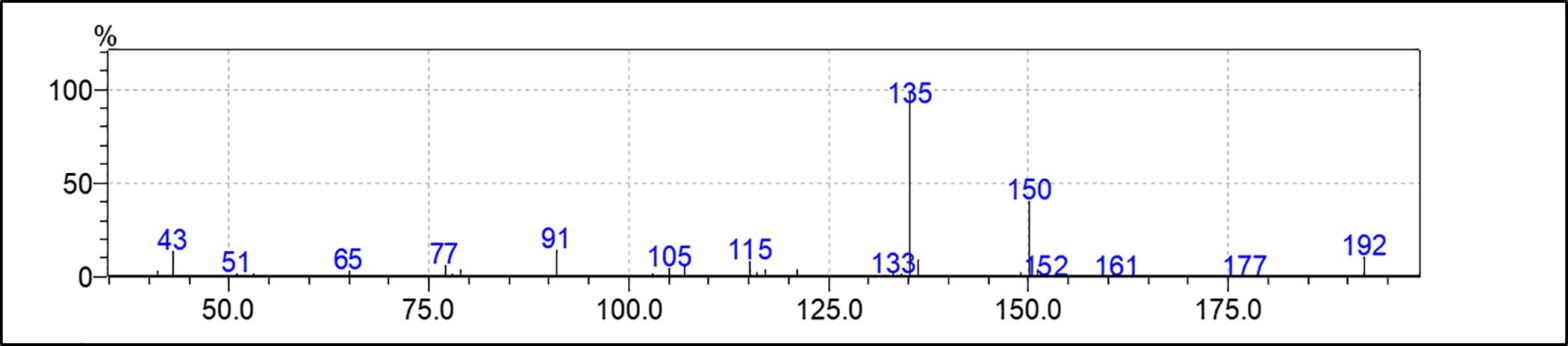
Thymol acetate mass spectrum.

Thymol acetate was obtained by the acetylation of thymol (Fig 1) in 99.38% purity. The chromatogram and mass spectrum of thymol acetate are shown in Figs 2 and 3.

### Anticholinesterase activity

The aim of this study is to contribute to the search for new inhibitors of AChE from natural sources. Thus, the anticholinesterase activity of the essential oil from the leaves of *L*. *thymoides* was investigated. In addition, we determined the activity of two of the main constituents of the oil, thymol and thymol acetate (99.38% purity).

The essential oil of the leaves of *L*. *thymoides* (thymol: 66.33%) showed a detection limit (DL) at a concentration of 0.1 ng/spot, about ten times less active than physostigmine (DL = 0.01 ng/spot). Because thymol and thymol acetate presented a DL values of 0.01 ng/spot, equivalent to that of physostigmine, the alkaloid was used as a positive control. The higher detection concentration of thymol and thymol acetate in the oil may be because these components have a lower total content in the sample mixture compared to the isolate. Thus, the anticholinesterase action of the essential oil of *L*. *thymoides* may be tied to these two components. These results are in agreement with data from the literature, which indicates that the essential oil of *Origanum ehrenbergii* (thymol: 19.6%) shows strong inhibitory activity against AChE, having a 50% inhibitory concentration (IC_50_) of 0.3 ± 0.02 μg/mL when physostigmine was used as positive control and exhibited an IC_50_equal to 0.1 ± 0.003 μg/mL [43]. The essential oil of *Thymus vulgaris* L. (thymol: 12%) showed an IC_50_ of 0.216 ± 0.011 mg/mL [44]. *O*. *vulgare* subsp. vulgare essential oil (thymol: 58.31%) was found to have a concentration of1.64 ± 0.002 mg galantamine equivalents per gram (GALAEs/g) [1].

Thymol and carvacrol have anticholinesterase activity reported by several authors, with different values of IC_50_: 0.74 mg/mL (thymol) and IC_50_ equivalent to 0.063 mg/mL (carvacrol) [45]; 0.212 ± 0.011 mg/mL (thymol) and IC_50_ 0.091 ± 0.011 mg/mL (carvacrol) [44]; 47.5 ± 1.08 mg/mL (thymol) and IC_50_ 182 ± 1.32 mg/mL (carvacrol) [46]. These studies have shown that the anticholinesterase activity of thymol is promising. Thus, the specimen of *L*. *thymoides* described in this work can be a natural source with potential for the development of new phytotherapics for the treatment of Alzheimer's disease or even be included in the diet of people with Alzheimer's. It is important to emphasize that in our previous work [47] it was shown that this specimen presents thymol as the majority constituent of essential oil throughout its seasonal cycle, which reinforces its viability as a source of obtaining this component.

### Interactions observed by molecular docking

Previously, molecular docking has been used to elucidate the interactions that occur between the ligands with the active site residues of AChE [4,48,49]. In the present study, molecular docking simulations were used to evaluate the complementarity and interactions between thymol and thymol acetate with the AChE binding site.

The binding energies, expressed by the MolDock scores obtained from molecular docking simulations, are shown in Table 2. The differences in energies can be explained by the difference in the molecular volume between thymol and thymol acetate.

**Table 2:** Results of docking energies obtained by the MolDock score.

Compound	MolDock score (kcal/mol)
Thymol	-72.82
Thymol acetate	-84.49

**Fig 4:**
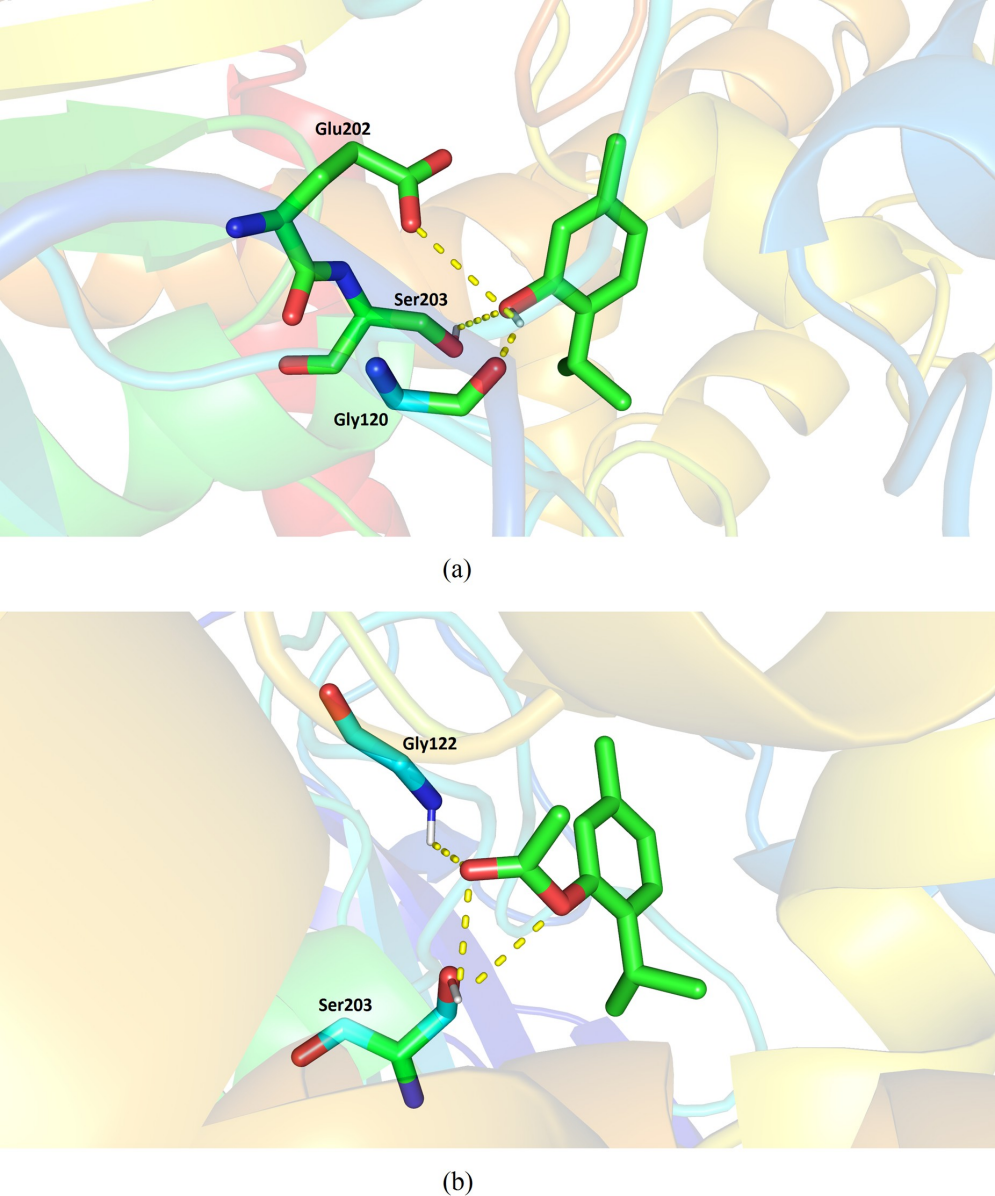
The result of molecular docking conformation obtained. (A) Molecular interactions for Thymol and (b) Thymol acetate in AChE binding pocket.

Thymol and thymol acetate form hydrogen bonding (H-bond) interactions with the Ser203 catalytic residue in the AChE binding pocket (Fig 4).

The O1 atom of the hydroxyl group, which belongs to thymol, forms a H-bond interaction with Ser203 at a distance of 3.14 Å. In contrast, thymol acetate forms two H-bond interactions with the Ser203 residue. The interactions with oxygen atom O2 of the Ser203 residue have distances of 2.66 and 2.98 Å.

Considering the catalytic mechanism of AChE, residues Ser203 and His447 are directly involved in covalent bond formation and breakage. Residue Ser203 is a nucleophilic site, whereas the His447 imidazole group acts as a catalytic base, accepting a proton transferred from Ser203 [50,51]. Thus, because molecular docking calculations have shown that thymol and thymol acetate interact with the same catalytic site residues of the enzyme, these inhibitors may show similar anti-AChE activity.

### Structural dynamics of the AChE systems

The structural dynamics of AChE complexed with the ligands thymol and thymol acetate during MD simulations were analyzed using the root mean square deviation (RMSD). The RMSD plots of the AChE structure were analyzed based on the Cα backbone, whereas the ligand structure was analyzed using the heavy atoms alone.

**Fig 5:**
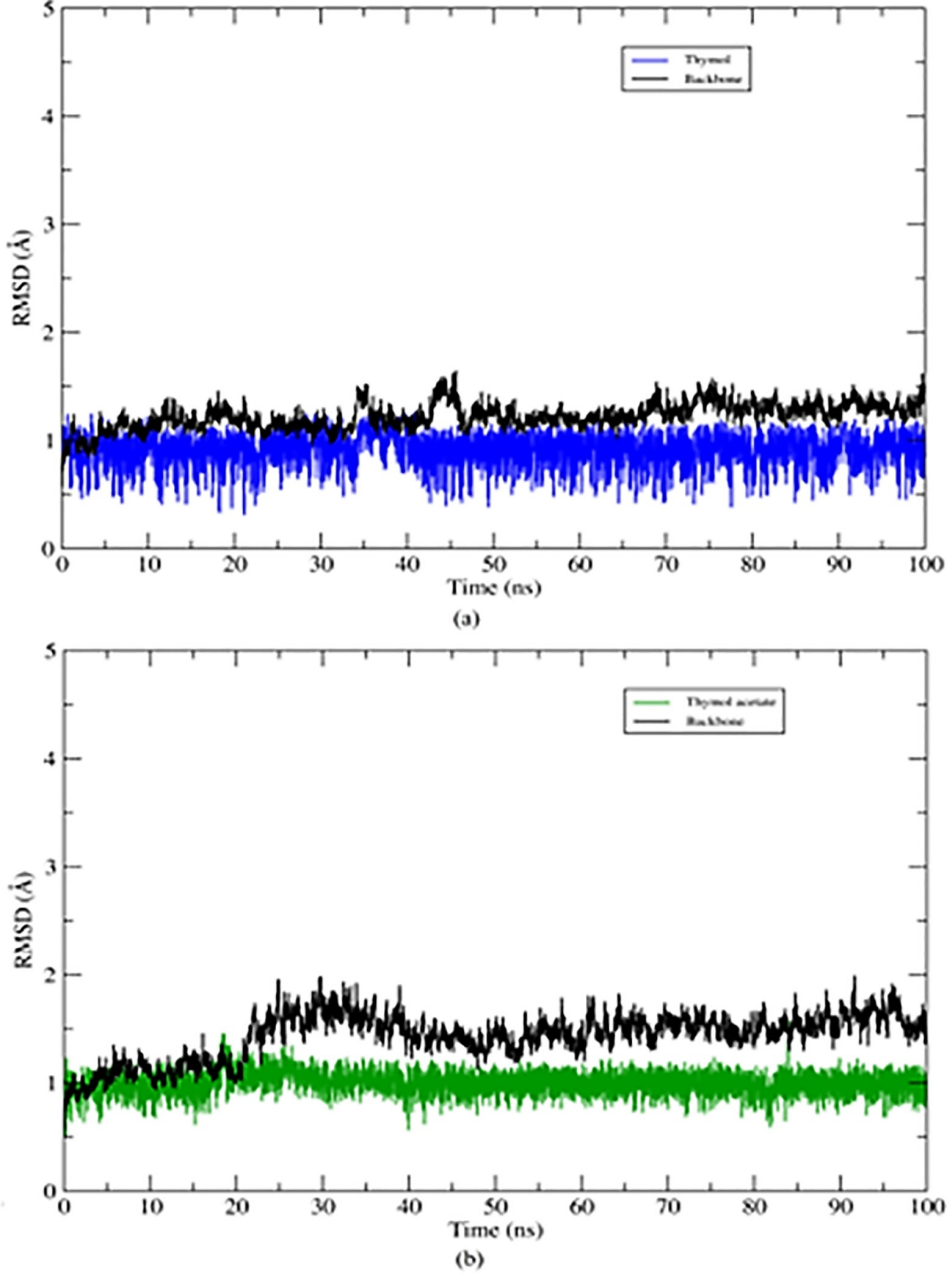
RMSD values for 100 ns of MD for AChE systems. The protein backbone is colored in black, thymol in blue and thymol acetate in green. (a) RMSD sistem plot Thymol- AChE e (b) RMSD sistem plot Thymol acetate-AChE.

The RMSD plot of AChE complexed to thymol and thymol acetate reaches a plateau (Fig 5), indicating that AChE structure is stable, showing few conformational changes over the 100-ns MD simulation. The ligands showed high stability at the AChE binding site. Over the 100-ns MD run, the ligands remained bound to AChE and did not undergo drastic conformational changes that altered their interactions with the catalytic residues.

### Binding free energy decomposition

Binding energy calculations show that thymol and thymol interact with high affinity in the AChE binding pocket, having Δ*G*_bind_ values of -18.49 and -26.88 kcal/mol, respectively. The binding free energy decomposition reveals that the van der Waals (Δ*E*_vdW_) contribution represents a large proportion of the interactions of the ligands in AChE binding pocket. The electrostatic (Δ*E*_electrostatic_) and non-polar (Δ*G*_SA_) contributions were also favorable for the formation of the complexes. The Δ*G* values and their components are listed in Table 3.

**Table 3:** Free energy variation (ΔG_bind_) and its components. ΔE_vdW_ represents the Van de Waals energy contribution, ΔE_ele_ represents the electrostatic energy, ΔG_GB_ polar contribution and ΔGSA non-polar contribution. All values are expressed in kcal/mol.

Compound	ΔE_vdW_	ΔE_ele_	ΔG_GB_	ΔG_SA_	ΔG_bind_
Thymol	-23.89	-7.97	16.47	-3.10	-18.49
Thymol acetate	-33.22	-14.11	24.52	-4.05	-26.88

### Intermolecular interactions analyses

**Fig 6:**
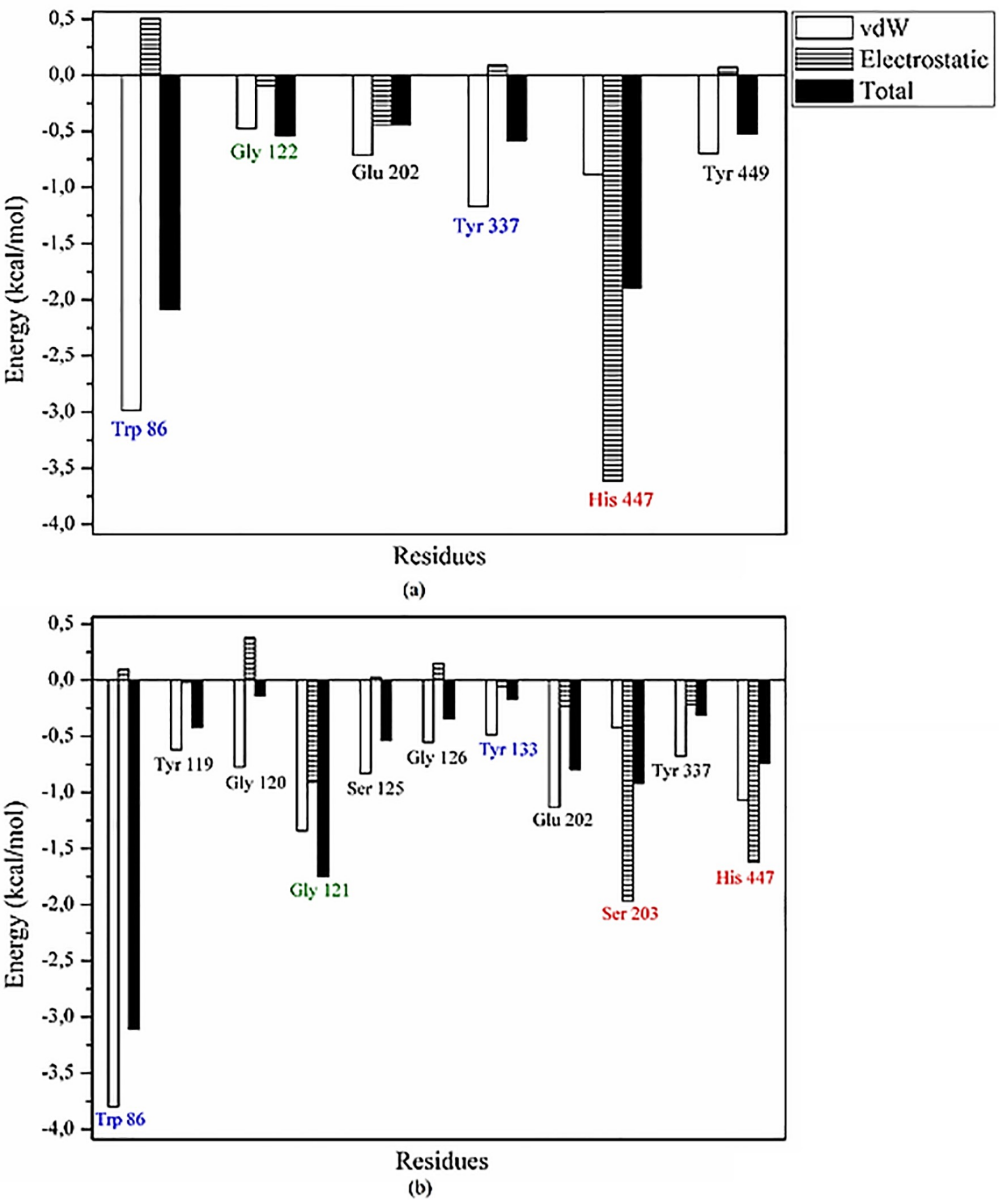
**Binding free energy decomposition for each residue that interact with (A) thymol and (B) thymol acetate.** The bars represent the energy contribution values: van der Waals contributions (blank bars), electrostatic contributions (striped bars) and total energy contribution for each residue (black bars). Residues highlighted in red are from catalytic site, blue are from anionic subsite and green are from oxyanion hole.

To explore the relative energy contribution to the overall ligand binding energy for each residue in the AChE binding pocket, the per-residue binding free energy decomposition was analyzed in Amber16. The results obtained are shown in Fig 6.

The AChE structure contains different binding sites [52]. The catalytic residues Ser203, Glu337, and His447 are located in the same cavity of the enzyme, which also contains other important residues for enzymatic activity, for example, Gly121, Gly122, and Ala204 (oxyanion hole), Trp86, Tyr133, Tyr337, and Phe338 (anionic subsite), and Phe295 and Phe297 (acyl pocket) [53].

Thymol acetate (Δ*G*_bind_ = -26.88 kcal/mol) has a better binding energy than that ofthymol (Δ*G*_bind_ = -18.49 kcal/mol); that is, the binding energy of thymol acetate is greater than that of thymol, and both the van der Waals and electrostatic energies are important for ligand binding in AChE cavity.

Thymol formed intermolecular interactions with different residues of the AChE binding pocket, in particular, with residue His447, which belongs to the catalytic triad (His 447, Tyr337, and Trp86) located on the anionic subsite. Residues His447 and Gly122 belong to the oxyanion hole. In contrast, thymol acetate formed different intermolecular interactions with AChE binding pocket and the electrostatic and van der Waals energy contributions were found to be significant in the interactions with residues Ser203 and His447 (catalytic triad), thus stabilizing ligand binding. Thymol acetate also formed important interactions with residues from two other sites, Trp86 and Tyr133 (anionic subsite) and Gly121 (oxyanion hole).

## Conclusions

Thymol is the main constituent in the essential oil from the flowers (37.86% DFL; 48.04% FFL), leaves (58.9% DLE; 66.33% FLE), and branches (63.59% FB; 66.2% DB) of *L*. *thymoides*, and the essential oil of the fresh leaves had the highest content of this oxygenated monoterpene. In the roots, hexadecanoic acid (palmitic acid) was the main constituent (38.02% DR; 40.92% DF) of the oil. The essential oil of the fresh leaves (DL = 0.1 ng/spot) and two of its mains components, thymol (DL = 0.01 ng/spot) and thymol acetate (DL = 0.01 ng/spot), showed inhibitory activity against acetylcholinesterase on TLC layers.

The MolDock scores obtained for thymol and thymol acetate were favorable. The poses obtained from molecular docking simulations showed that the ligands forms H-bond interactions with the Ser203 residue, which belongs to the catalytic triad. The RMSD values obtained over 100 ns of MD simulation showed that the ligands are stable in the AChE binding pocket. The binding free energies for thymol (Δ*G*_bind_ = -18.49 kcal/mol) and thymol acetate (Δ*G*_bind_ -26.88 kcal/mol) indicate the ligands are stable and bind with affinity to AChE. The per-residue binding free energy decomposition revealed that the ligands form interactions with residues that are important for catalytic activity. Some residues belonging to the catalytic triad (Ser203 and His447), anionic subsite (Trp86, Tyr333, and Tyr337), and oxyanion hole (Gly121 and Gly122) form intermolecular interactions that stabilize ligand binding.

## Supporting information

S1 Fig ion chromatogram relative to the essential oil composition of leaves and flowers of *Lippia thymoides*. (DOCX)

S2 Fig ion chromatogram relative to the essential oil composition of branches and roots of *Lippia thymoides*. (DOCX)
