# Supplementary material for: Chemical profile of Lippia thymoides, evaluation of the acetylcholinesterase inhibitory activity of its essential oil, and molecular docking and molecular dynamics simulations
Source: PLoS One. 2019 Mar 8;14(3):e0213393. doi: 10.1371/journal.pone.0213393 (PMC6407782; doi:10.1371/journal.pone.0213393)
Supplement: S1 Fig — (DOCX) [file pone.0213393.s001.docx]

**S1 Fig. ion chromatogram relative to the essential oil composition of leaves and flowers of *Lippia thymoides***

***
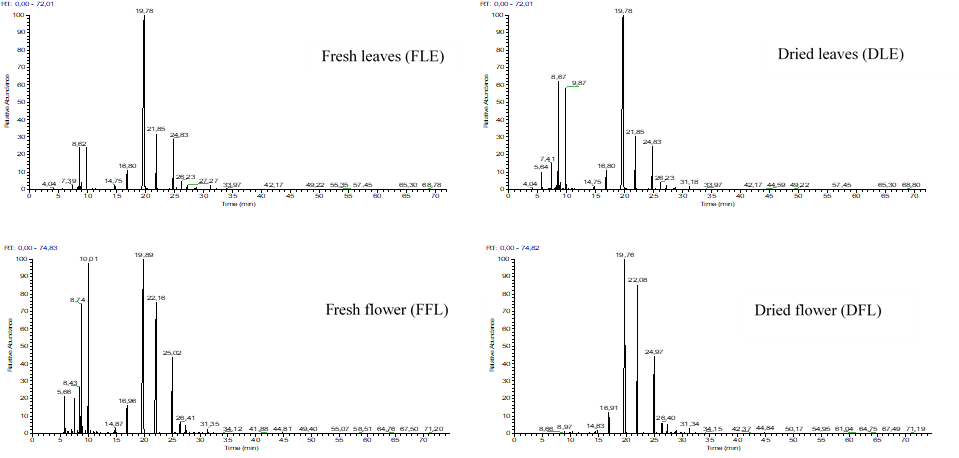
***
